# The Photosensitizer Octakis(cholinyl)zinc Phthalocyanine with Ability to Bind to a Model Spike Protein Leads to a Loss of SARS-CoV-2 Infectivity In Vitro When Exposed to Far-Red LED

**DOI:** 10.3390/v13040643

**Published:** 2021-04-09

**Authors:** Kirill Sharshov, Mariya Solomatina, Olga Kurskaya, Ilya Kovalenko, Ekaterina Kholina, Vladimir Fedorov, Gennady Meerovich, Andrew Rubin, Marina Strakhovskaya

**Affiliations:** 1 Federal Research Center of Fundamental and Translational Medicine (CFTM), 630117 Novosibirsk, Russia; sharshov@yandex.ru (K.S.); mariaza@ngs.ru (M.S.); kurskaya_og@mail.ru (O.K.); 2 Faculty of Biology, Lomonosov Moscow State University, 119234 Moscow, Russia; ikovalenko78@gmail.com (I.K.); tenarra1@gmail.com (E.K.); xbgth@yandex.ru (V.F.); rubin@biophys.msu.ru (A.R.); 3 Federal Scientific and Clinical Center of Specialized Types of Medical Care and Medical Technologies of the Federal Medical and Biological Agency of Russia, 115682 Moscow, Russia; 4 Prokhorov General Physics Institute of the Russian Academy of Sciences, 119991 Moscow, Russia; meerovich@mail.ru; 5 Institute of Physics and Engineering in Biomedicine, National Research Nuclear University “MEPHI”, 115409 Moscow, Russia

**Keywords:** SARS-CoV-2, spike protein, photodynamic inactivation, octakis(cholinyl)zinc phthalocyanine, LED, Brownian dynamics

## Abstract

Photodynamic inactivation of pathogenic microorganisms can be successfully used to eradicate pathogens in localized lesions, infected liquid media, and on various surfaces. This technique utilizes the photosensitizer (PS), light, and molecular oxygen to produce reactive oxygen species that kill pathogens. Here, we used the PS, water soluble octakis(cholinyl)zinc phthalocyanine (Zn-PcChol_8+_), to inactivate an initial 4.75–5.00 IgTCID50/mL titer of SARS-CoV-2, thereby preventing viral infection when tested in Vero E6 cell cultures. Zn-PcChol_8+_ in a minimally studied concentration, 1 µM and LED 3.75 J/cm^2^, completely destroyed the infectivity of SARS-CoV-2. To detect possible PS binding sites on the envelope of SARS-CoV-2, we analyzed electrostatic potential and simulated binding of Zn-PcChol_8+_ to the spike protein of this coronavirus by means of Brownian dynamics software, ProKSim (Protein Kinetics Simulator). Most of the Zn-PcChol_8+_ molecules formed clusters at the upper half of the stalk within a vast area of negative electrostatic potential. Positioning of the PS on the surface of the spike protein at a distance of no more than 10 nm from the viral membrane may be favorable for the oxidative damage. The high sensitivity of SARS-CoV-2 to photodynamic inactivation by Zn-PcChol_8+_ is discussed with respect to the application of this PS to control the spread of COVID-19.

## 1. Introduction

Severe acute respiratory syndrome coronavirus 2 (SARS-CoV-2) is a lipid-enveloped single-stranded (+)RNA virus in the *Betacoronavirus* genus in the *Coronaviridae* family. In the last 20 years, *Betacoronavirus* have been in the spotlight due to outbreaks of severe acute respiratory syndrome (SARS-CoV) in 2002–2003 [1], Middle East respiratory syndrome (MERS-CoV) in 2012 [2], and the current COVID-19 pandemic (SARS-CoV-2) [3].

Human coronaviruses, including SARS-CoV-2, are released into the environment in respiratory droplets when an infected person coughs, sneezes, or talks. Viruses that are contained in the droplets can infect other people through the respiratory tract if they do not maintain a safe distance, or if they enter their body indirectly [4,5]. One of the indirect routes is through contaminated surfaces. Recent studies have shown that human coronaviruses can persist on various surfaces at room temperature for up to 9 days [6]. The existence of coronaviruses in water, sewage, and wastewater in a few days’ time frame is also possible and discussed in detail in the review [7]. Thus, the disinfection of repeatedly touched surfaces and decontamination of water/wastewater is essential to interrupt the possible transmission routes of these pathogens.

Many photosensitizers (PSs) involved in photodynamic reactions can cause effective damage to the enveloped viruses, and therefore, PSs are considered promising agents to combat with SARS-CoV-2 [8,9]. However, a limited number of experimental studies used PSs to inactivate coronaviruses. The photodynamic disinfection of blood plasma from SARS-CoV in the THERAFLEX MB-Plasma system was successful with a standard dose of methylene blue (0.085 mg per 250 mL of plasma) and visible light doses (30–120 J/cm^2^); the SARS-CoV titers were reduced by about three orders of magnitude, compared to the level of the other two tested viruses (arbovirus Congo-Crimean hemorrhagic fever and paramyxovirus Nipah) [10]. In suspensions with virus titers 3.0–4.0 lgTCID50, SARS-CoV-2 was inactivated using 1.0–10.0 μg/mL methylene blue, or 0.5–5.0 μg/mL Radachlorin, and 662 nm irradiation (16 and 40 J/cm^2^) by a semiconductor laser [11]. However, both PSs, methylene blue and Radachlorin, are able to inhibit the replication of SARS-CoV-2 at a very low micromolar range even without irradiation [11,12] that makes it difficult to assess the true photodynamic effect.

Cationic water-soluble zinc phthalocyanines with a high quantum yield of singlet oxygen production are powerful photoantimicrobials against a broad spectrum of pathogens, including enveloped viruses [13,14,15,16,17,18,19,20]. In this study, we used an octacationic derivative, octakis(cholinyl)zinc phthalocyanine (Zn-PcChol_8+_), and LED with the emission maximum at 692 nm to inactivate infectivity of SARS-CoV-2 tested in Vero E6 cell cultures. Due to the presence of eight positively charged peripheral substituents in the phthalocyanine molecule, electrostatic interactions should play an essential role in determining the properties of this PS; electrostatic repulsion results in a monomeric state of Zn-PcChol_8+_ in aqueous solutions, and electrostatic attraction—in its binding to the negatively charged bacterial cells [16]. In order to reveal the possible binding sites of the PS on the envelope of SARS-CoV-2, we analyzed the electrostatic potential fields of the S-protein of this coronavirus and Zn-PcChol_8+_ molecule and studied interactions of these molecules by Brownian dynamics (BD).

## 2. Materials and Methods

### 2.1. Cell Culture

Vero E6 cells (monkey kidney cells) were grown in Dulbecco’s Modified Eagle Medium (DMEM, Capricorn Scientific, Ebsdorfergrund, Germany) supplemented with 10% fetal bovine serum (FBS, Capricorn Scientific, Germany) and 50 µg/mL gentamicin sulfate (Biolot, St. Petersburg, Russia) at 37 °C under 5% CO_2_. After virus inoculation, cells were cultured in DMEM supplemented with 2% FBS and 50 µg/mL gentamicin sulfate. In all experiments, cells that were not infected with the virus were included as controls.

### 2.2. Virus Propagation, Purification and Titration

All SARS-CoV-2 experiments were performed according to the standard protocols at the BSL 3 Laboratory (Federal Research Center of Fundamental and Translational Medicine (CFTM), Novosibirsk, Russia).

Coronavirus SARS-CoV-2 strain hCoV-19/Novosibirsk/3886/2020 was isolated from a patient with COVID-19 and propagated in Vero cells grown in Dulbecco’s Modified Eagle Medium (DMEM/F12, Capricorn Scientific, Ebsdorfergrund, Germany) supplemented with 10% fetal bovine serum (FBS-52A, Capricorn Scientific, Germany) at 37 °C under 5% CO_2_. Virus stock was harvested and filtrated through a 0.22 µm filter and stored at −80 °C until use.

For virus titration, stocks of treated or non-treated viruses were serially diluted 10-fold starting from 10^−1^ to 10^−6^. Confluent monolayers of Vero cells, grown in 96-well plates, were washed with PBS and infected with respective dilutions of each virus. After 40 min of incubation at 37 °C, the supernatant was removed, and 200 µL of virus growth medium was added per well. Plates were incubated at 37 °C under 5% CO_2_ for 7 days. All virus titrations were performed in triplicate. Virus-induced cytopathic effect was detected, and virus titers were determined as the 50% tissue culture infectious dose (TCID50) per mL, according to Reed and Muench [21]. The photos were obtained using the EVOS XL Core Imaging System (ThermoFisher Scientific, Waltham, MA, USA), phase contrast mode 4/10, magnification ×20.

### 2.3. Photodynamic Inactivation

**Figure 1:**
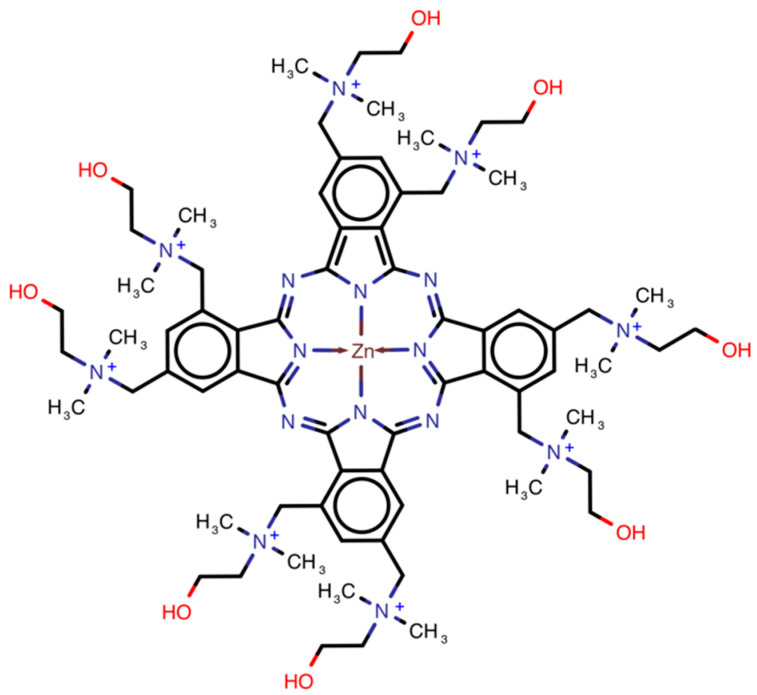
Molecular structure of Zn-PcChol_8+_.

**Figure 2:**
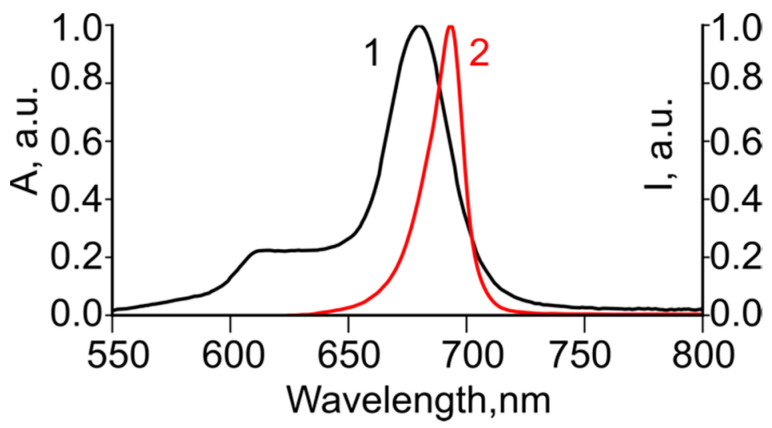
Normalized absorption spectrum of Zn-PcChol_8+_ in water (1) and emission spectrum of light emitting diode source (2).

Photosensitizer octakis(cholinyl)zinc phthalocyanine (Zn-PcChol_8+_), synthesized in the State Scientific Center “NIOPIK” (Russia) [22], was used for virus inactivation at concentrations of 1–5 µM. The molecular structure of Zn-PcChol_8+_ is shown in Figure 1. The irradiation system was built on six light-emitting diodes supplied with heat removal. Its emission spectrum, with a spectral maximum at 692 nm, was close to the absorption spectrum of Zn-PcChol_8+_ (Figure 2). Virus suspensions (200 µL of harvested virus stock in the growth medium filtrated through a 0.22 µm filter) were irradiated with or without (light control) Zn-PcChol_8+_ in square 1 × 1 cm plastic cuvettes for 2 or 5 min, at light doses of 1.50 or 3.75 J/cm^2^, respectively. The intensity of light reaching the probes was measured with PM160T Wireless Power Meter (THORLABS GmbH, Dachau, Germany) and made up 12.5 mW/cm^2^. The experiments were carried out in triplicate.

### 2.4. Brownian Dynamics Simulations and Cluster Analysis

For the Zn-PcChol_8+_ molecule Brownian diffusion simulation and its long-range electrostatic interaction with SARS-CoV-2 spike protein, we used our rigid body BD software “ProKSim” (Protein Kinetics Simulator, [23,24]). A three-dimensional model of the protein molecule was adopted from [25]. The model of the Zn-PcChol_8+_ molecule was adopted from [26].

In the BD simulation, the SARS-CoV-2 spike protein molecule was represented as a low dielectric area (ε = 2) with spatially fixed partial charges. The solvent was considered a high dielectric area (ε = 80) with mobile charges (ions), implicitly described through the ionic strength. In the simulation, electrostatic interactions were taken into account only when molecules approached 3.5 nm and closer. A Poisson–Boltzmann calculation [27] was used to determine the electrostatic potential grid around each molecule, as earlier described in detail [28].

Initially, the Zn-PcChol_8+_ molecule was placed in a random position and orientation in the reaction volume. The BD simulation was then continued until attractive electrostatic energy reached a given threshold value, and the obtained structure of electrostatic encounter complex was saved for further analysis. For each threshold value, ten thousand BD simulations with various initial positions were performed.

To analyze the relative positions of the Zn-PcChol_8+_ molecules in the ensemble of configurations obtained by the BD simulation for classifying structures, we used a single parameter hierarchical method that utilizes the density-based clustering algorithm [24,29]. A cluster of structures is considered a dense group that is separated from the other adjacent groups by less dense regions. For each cluster, we selected the so-called central structure having the minimum average root mean square deviation distance from all the other structures in the cluster.

## 3. Results

### 3.1. Photodynamic Inactivation of SARS-CoV-2

In the case of viruses, photodynamic inactivation can be defined as a decrease in a median tissue culture infective dose (TCID50), which means a loss of the ability to infect host cells and destroy 50% of the cells in monolayers. In our study, virus titers were determined using the Vero E6 cells culture; the initial titers were 4.75–5.00 lgTCID50/mL.

Four groups of SARS-CoV-2 suspensions were examined for infectivity, namely:

1. Suspension without any treatment (control).
2. No irradiated suspensions after 10 min incubation with 1, 2, or 5 µM of Zn-PcChol_8+_ (dark controls).
3. Suspensions irradiated at doses of 1.50 J/cm^2^ or 3.75 J/cm^2^, respectively, for 2 or 5 min, without the PS (light controls).
4. Suspensions irradiated at doses of 1.50 J/cm^2^ or 3.75 J/cm^2^, respectively for 2 or 5 min, and each was incubated for 10 min before irradiation with 1, 2, or 5 µM of Zn-PcChol_8+_.

The SARS-CoV-2 titers in the control groups 1 and 2 were similar (Table 1), thus indicating the absence of Zn-PcChol_8+_ toxicity toward SARS-CoV-2 in the range of 1–5 µM in the dark. Irradiation with far-red LED light in the absence of the PS (group 3) had no noticeable effect on virus titer as expected.

**Figure 3:**
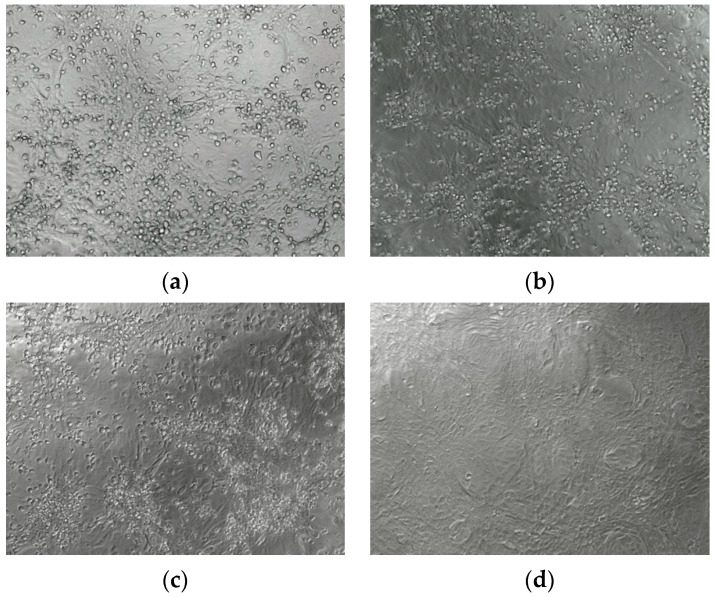

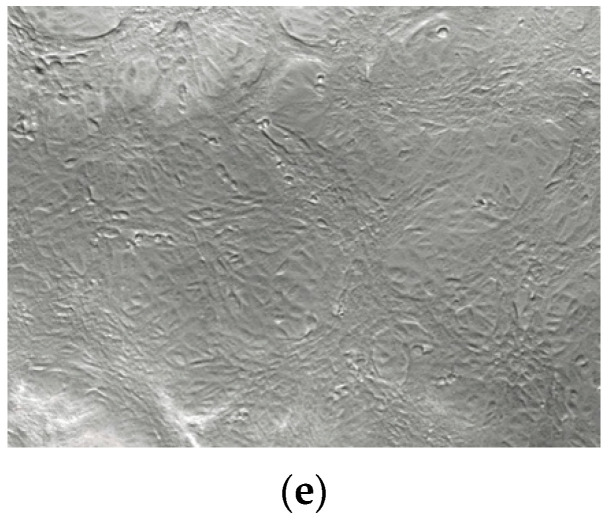
The monolayers of Vero 6 cells 48 h after infection with SARS-CoV-2 untreated (**a**), treated with 1 µM Zn-PcChol_8+_ (**b**), irradiated with 3.75 J/cm^2^ LED light at 692 nm (**c**), treated with 1 µM Zn-PcChol_8+_ and irradiated with LED 3.75 J/cm^2^ (**d**), and uninfected Vero E6 monolayer (**e**). Photographs were made with magnification ×20.

In group 4, LED irradiation for 2 min (1.50 J/cm^2^) resulted in a complete loss of SARS-CoV-2 infectivity in the presence of 2 or 5 µM Zn-PcChol_8+_ and a partial decrease of virus titers with 1 µM Zn-PcChol_8+_ (Table 1). In the samples incubated with Zn-PcChol_8+_ (1, 2, or 5 µM) and irradiated with LED for 5 min (3.75 J/cm^2^), we found complete protection of Vero E6 cells from lyses, that is, complete loss of virus infectivity (Table 1). This effect may be associated with photodynamic inactivation since Zn-PcChol_8+_ in concentrations up to 5 µM in the absence of irradiation, as well as only LED 692 nm in the studied doses, did not affect SARS-CoV-2 titers. Figure 3 shows the photographs of Vero E6 cells monolayers: (a) infected with SARS-CoV-2; (b) infected with SARS-CoV-2 and treated with 1 µM Zn-PcChol_8+_; (c) infected with SARS-CoV-2 and irradiated with LED 3.75 J/cm^2^; (d) infected with SARS-CoV-2 and treated with 1 µM Zn-PcChol_8+_ and irradiated with LED 3.75 J/cm^2^; (e) uninfected monolayers. We confirmed the complete loss of virus infectivity by negative second passage in Vero E6 cells.

### 3.2. Electrostatic Fields of PS Molecule and SARS-CoV-2 Spike Protein, Brownian Dynamics Simulations and Cluster Analysis

It has long been postulated that antiviral PSs must bind to the viral lipid envelope (in the case of enveloped viruses), or the protein coat, or the genome [30]. In coronaviruses, the envelope contains a lipid bilayer with at least three embedded viral proteins: the membrane protein (M-protein) and the envelope protein (E-protein) are involved in virus assembly, while the spike protein (S-protein) mediates virus entry into the host cells [31]. The most abundant M-proteins and E-proteins are transmembrane proteins with short (19 and 16 amino acid residues, respectively) N-terminal ectodomains; in contrast, each ectodomain of the trimeric S-protein contains 1213 amino acid residues out of a total of 1273 [32]. Unsaturated fatty acids of viral lipids are susceptible to singlet oxygen generated by the membrane-binding PSs, such as the lipophilic thiazolidine derivative [33]. However, intercalating of Zn-PcChol_8+_ in the viral lipid bilayer that originates from the host cell membranes seems unlikely since the octacationic Zn-PcChol_8+_ molecules showed no affinity to the POPC model bilayer in our recent simulation experiments [26]. Moreover, Zn-PcChol_8+_ molecules almost never appeared at distances less than 3.5 nm from the POPC bilayer center.

**Figure 4:**
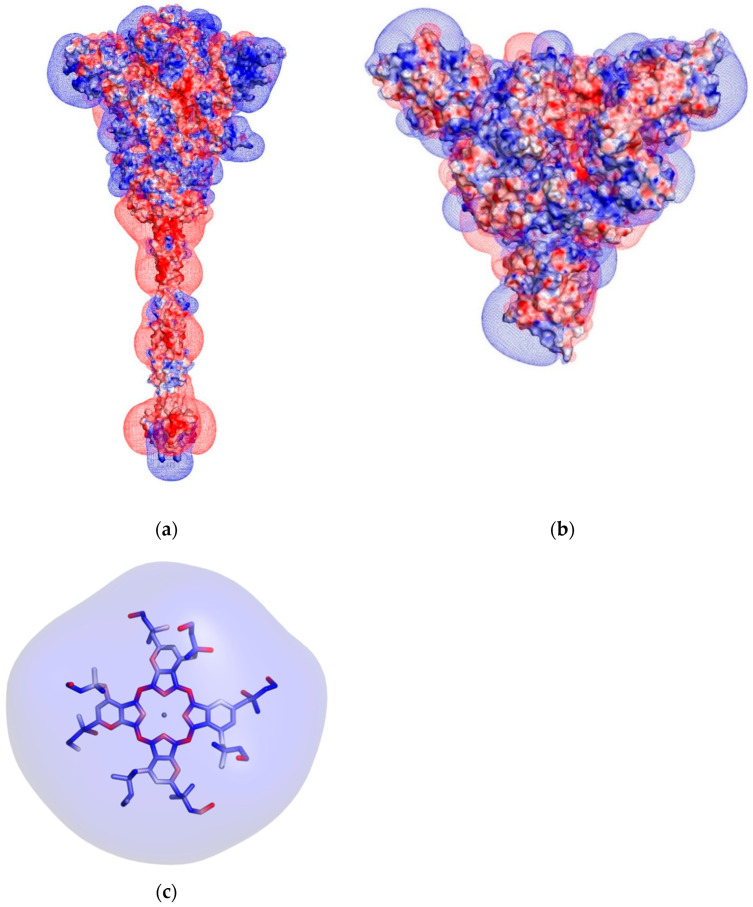
Distribution of electrostatic potential on the molecular surface of the S-protein trimer colored from red (–100 mV) to blue (+100 mV) and its equipotential electrostatic surfaces represented by red mesh (–7 mV) and blue mesh (+7 mV) in the lateral view (**a**) and top view (**b**), and stick model of Zn-PcChol_8+_ (**c**) colored by molecular surface electrostatic potential from –100 mV (red) to +100 mV (blue). Ionic strength is 100 mM.

Can ectodomains of the S-protein that protrude from the viral membrane provide binding sites for the Zn-PcChol_8+_ molecules? To answer the question, we first calculated the electrostatic potential field of the S-protein and Zn-PcChol_8+_ according to Poisson–Boltzmann formalism. Figure 4 displays the surface distribution of electrostatic potential and equipotential electrostatic surfaces of SARS-CoV-2 spike protein (a) and (b), and Zn-PcChol_8+_ (c) at ionic strength 100 mM. It is easy to see that while the Zn-PcChol_8+_ molecule is mostly positively charged, the S-protein stalk shows three large areas of negative electrostatic potential, with the largest negatively charged area located at the conjunction of the stalk and the head of the spike protein. The head itself is mostly positive, though several patches of negative potential are present. From panel (b), one can also see that the distribution of electrostatic potential is not strictly symmetric.

**Figure 5:**
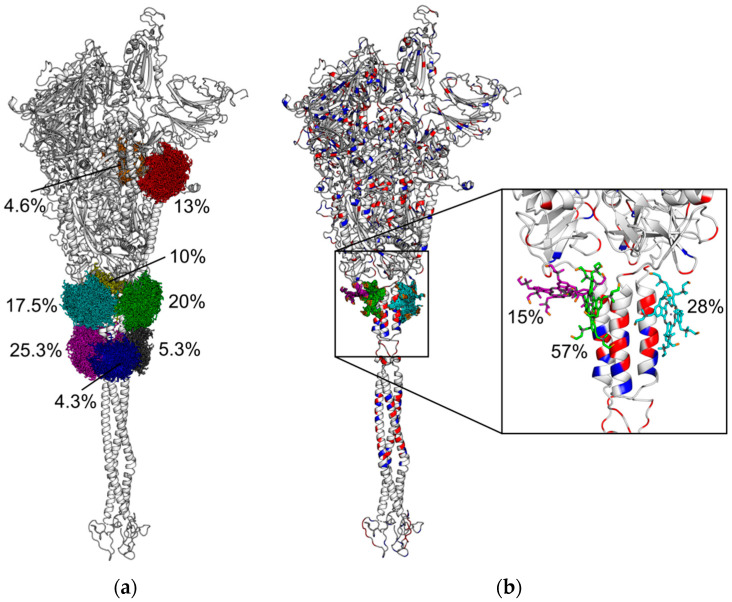
Cartoon representation of SARS-CoV-2 spike protein with clusters of Zn-PcChol_8+_ molecules with electrostatic energy of attraction to SARS-CoV-2 spike protein of more than 8 *kT* (**a**) and 11 *kT* (**b**). The structures of Zn-PcChol_8+_ molecules belonging to the same cluster are colored by the same color. The relative sizes of clusters, in percentage terms, are given in the figure. The secondary structure of protein in plate (**b**) is colored by the type of amino acid charge residues (blue are Lys and Arg residues, and red are Glu and Asp residues). The inset shows the magnified area of SARS-CoV-2 spike protein with central structures of three clusters revealed by cluster analysis at attraction electrostatic energy of more than 11 *kT*.

Since the Zn-PcChol_8+_ molecules and certain areas of the spike protein of SARS-CoV-2 have opposite charges, their electrostatic interaction is most likely. We analyzed the possible structures of Zn-PcChol_8+_ with the spike protein of SARS-CoV-2 complexes generated by means of BD with account of electrostatic interactions by software, ProKSim. It turned out that Zn-PcChol_8+_ molecules formed encounter complexes with electrostatic attraction energy exceeding 8 *kT* at certain sites on the surface of the spike protein, as shown in Figure 5a. We applied density-based hierarchical cluster analysis to study the variety of encounter complex structures. Cluster analysis revealed eight ensembles of energetically favorable structures of Zn-PcChol_8+_ molecules in encounter complexes with the spike protein, shown in Figure 5a in different colors. One can see that most of the Zn-PcChol_8+_ molecules (82.4% of all structures) formed six clusters, positioned around the upper half of the stalk where a vast area of negative electrostatic potential is located, attracting positively charged PS molecules. The rest of the structures (17.6%) formed two clusters, positioned at two large areas of negative electrostatic potential at the head of the spike protein of SARS-CoV-2.

The subsequent BD simulations with 11 *kT* threshold allowed finding the most energetically favorable positions of Zn-PcChol_8+_ molecules, with respect to SARS-CoV-2 S-protein. In this case, cluster analysis revealed only three clusters (Figure 5b) of energetically favorable structures. All PS molecules were positioned at the conjunction of the stalk and the head of the spike protein of SARS-CoV-2, where a lot of negatively charged amino acid residues could be found. The inset figure shows the magnified image of this area containing central structures of the three clusters. We found that at electrostatic attraction energy of no more than 8 *kT*, electrostatic interactions allowed PS molecules to rotate almost freely around their own mass center in the vicinity of negatively charged amino acid residues at the spike protein, while at 11 *kT* energy, only certain orientations of PS molecules were possible. Interestingly, the encounter complexes with electrostatic attraction energy exceeding 12 *kT* have not been found, even in a millisecond simulation.

## 4. Discussion

We suggest that the stable structures of Zn-PcChol_8+_ and spike protein electrostatic complexes obtained in our BD simulations may be favorable to provide photodynamic damage to nearby located sensitive viral structures. In fact, as we have found, positively charged Zn-PcChol_8+_ molecules concentrate near negatively charged amino acid residues of the SARS-CoV-2 spike protein ectodomains (Figure 4 and Figure 5). It is well established that, in comparison with SARS-CoV, the surface of SARS-CoV-2 S-protein exhibits a more positive electrostatic potential [34]. However, while its S-protein head carries a positive electrostatic potential, the stalk, according to the previous data [34] and our simulations (Figure 4), is negatively charged and provides the appropriate surface encounter to the Zn-PcChol_8+_ molecules. Our BD simulations have demonstrated that the most stable binding occurs at the junction of the stalk with the head of the spike protein at a distance of about 10 nm from the intramembrane domain. Zn-PcChol_8+_ is the Type II PS, with a high quantum yield of singlet oxygen (^1^O_2_) production (0.65) [22]. In the aqueous medium, the lifetime of ^1^O_2_ is about 4 μs but is shortened in a biological environment. In cells, the lifetime of ^1^O_2_ is in the range of 10–320 ns, and its diffusion distance is 10–55 nm [35]. According to the authors [36], ^1^O_2_ has an even more limited lifetime and diffusion distance, 40 ns and 20 nm, respectively. Thus, for effective oxidative damage, the PS should be located at a distance of no more than the diffusion radius of ^1^O_2_ from the sensitive biological structures. In terms of antimicrobial photodynamic treatment, this means that the adhesion of the PS to the external structures of the targeted pathogen should be sufficient to allow its destruction [37]. Our BD simulations show (Figure 5) that Zn-PcChol_8+_ binds to SARS-CoV-2 S-protein at a distance of no more than 10 nm from the viral membrane. Surface proteins and the lipid bilayer are both sensitive to oxidative damage. In fact, we have shown that Zn-PcChol_8+_ causes photodynamic inactivation of avian influenza A viruses of the H5N1 [15] and H5N8 [20] subtypes. Three different types of H5N8 damage were observed after treatment with 4 µM of Zn-PcChol_8+_ and 12 J/cm^2^ of white light [20], namely, “bald” virions with removed surface glycoproteins and membranes retaining structural integrity, virions with damaged membranes, and totally destroyed virions, all forms being non-infectious. 

Our simulation data show that Zn-PcChol_8+_ binds mainly to S2 subunit of SARS-CoV-2 spike protein at the junction of spike head and stalk. Since this site is not directly involved in the interaction with ACE2 receptor, binding of Zn-PcChol_8+_ does not appear to affect SARS-CoV-2 attachment and the infecting of the host cell. Our experimental data confirm the lack of Zn-PcChol_8+_ antiviral activity in the absence of irradiation. Indeed, Zn-PcChol_8+_ itself did not affect SARS-CoV-2 titer and only under irradiation caused the loss of infectivity, that is, virus photodynamic inactivation (Table 1). This distinguishes Zn-PcChol_8+_ from methylene blue, a tricyclic phenothiazine dye, whose antiviral activity is observed even without photoactivation. Methylene blue inhibits SARS-CoV-2 attachment and entry by blocking the protein–protein interactions of the S-protein receptor binding domain, located in S1 subunit, with ACE2 on the host cell [38].

Earlier, phthalocyanine Zn(II) derivatives were proposed to be used as virus inactivating agents for photodynamic disinfection of surfaces, instruments, and biological fluids in the hospital hygiene strategies [39], as well as for photodynamic disinfection of different aqueous media [15]. In this study, the octacationic phthalocyanine Zn(II) derivative (Zn-PcChol_8+_), in a minimal concentration of 1 µM under 692 nm LED light irradiation at a dose of only 3.75 J/cm^2^, completely inactivated SARS-CoV-2 with the titer 5.00 lgTCID50/mL in the medium rich in inorganic salts and proteins, similar in composition to saliva droplets. The high sensitivity of SARS-CoV-2 to Zn-PcChol_8+_-induced photodynamic inactivation makes this PS promising for disinfection purposes to combat the spread of COVID-19.

## Figures and Tables

**Table 1:** SARS-CoV-2 titers (lgTCID50/mL) by virus titration on Vero E6 cells.

Concentration of Zn-PcChol_8+_, µM	Dose of LED Light at 692 nm, J/cm^2^		
	0	1.50	3.75
0	4.83 ± 0.17	4.38 ± 0.38	4.92 ± 0.04
1	5.00 ± 0.07	2.90 ± 0.07	0
2	4.88 ± 0.19	0	0
5	4.75 ± 0.13	0	0

## Data Availability

Not applicable.
